# Usefulness of the Rapid Office Strain Assessment (ROSA) tool in detecting differences before and after an ergonomics intervention

**DOI:** 10.1186/s12891-022-05490-8

**Published:** 2022-06-02

**Authors:** Fernanda Cabegi de Barros, Cristiane Shinohara Moriguchi, Thaís Cristina Chaves, David M. Andrews, Michael Sonne, Tatiana de Oliveira Sato

**Affiliations:** 1 Physical Therapy Department, Laboratory of Preventive Physical Therapy and Ergonomics (LAFIPE), Federal University of São Carlos, Rodovia Washington Luís, km 235, Monjolinho, São Carlos, São Paulo, 13565-905 Brazil; 2 Department of Kinesiology, University of Windsor, Windsor, ON Canada; 3 Occupational Health Clinics for Ontario Workers Inc, Hamilton, ON Canada

**Keywords:** Musculoskeletal discomfort, Prevention & control, Occupational health

## Abstract

**Background:**

Most ergonomics studies on office workstations evaluate the effects of an intervention only by subjective measures such as musculoskeletal pain and discomfort. Limited evidence has been provided regarding risk factor reduction in office environments through standardized methods assessments. The Rapid Office Strain Assessment (ROSA) tool can provide an estimation of risk factor exposure for office workers as a means by which the outcome of interventions can be quantified.

**Purpose:**

The aim of the study was to evaluate if ROSA scores reflect changes in risk factors after an ergonomics intervention among office workers.

**Methods:**

Office workers (*n* = 60) were divided into two groups. The experimental group received a workstation intervention and the control group received no intervention. Changes in ROSA scores were compared before and after the intervention in both groups.

**Results:**

Statistically significant reductions in the ROSA final and section scores occurred after the intervention in the experimental group with (mean reduction of 2.9, 0.8 and 1.6 points for sections A, B and C, respectively). In contrast, no differences were detected in the control group (mean increase of 0.1 point for sections A and C and mean reduction of 0.1 point for Section B).

**Conclusions:**

These findings show that ROSA scores reflect changes in risk factors after an ergonomics intervention in an office environment. Consequently, this tool can be used for identifying and controlling risk factors among computer workers, before and after interventions.

## Background

Sitting continuously for long periods in awkward postures, with unadjusted monitor heights, with mice positioned far from the body, and with unadjusted chairs are prominent risk factors for neck/shoulder, back and arm pain among office workers [1–3]. These risk factors have become even more frequent in the last year, due to the COVID-19 pandemic, which increased the amount of time many workers spent on desktop/laptop computers during lockdown [4].

The prevalence of neck/shoulder and low back pain is high among office workers; therefore, interventions in which workers are advised to adjust their workstation to reduce musculoskeletal symptoms are common [5–7]. However, systematic reviews have identified a lack of standardized methods for measuring the effectiveness of these interventions to reduce risk factors in the workplace [8–10]. Moreover, evidence about exposure changes after interventions remains limited [10], and may be due to the fact that most studies to date have evaluated the effects of interventions using subjective measures such as musculoskeletal pain and discomfort [10–12]. Thus, postural risk assessment, by means of a precise, accurate and sensitive tool, is very relevant to measure any reduction in risk that may be achieved by ergonomics interventions and, consequently, to strengthen the evidence of ergonomic intervention effectiveness.

The use of kinematic evaluation by means of motion capture systems, such as cameras and wearable systems, has been essential for human-centered design innovation in the workplace and for reducing workplace risk factors [13]. Using 3D images and wearable systems may be more accurate than observational or 2D images for work assessment and modifying working conditions. Advances in low cost motion capture systems have been already reported [14]. However, these systems are still time consuming to use, and require financial investment and practitioner training, which may not be accessible for all companies, mainly in low-income countries.

Measurement of postural risks can be accomplished using standardized observation-based protocols such as the Ovako Working posture Assessment System (OWAS), Quick Exposure Check (QEC), Rapid Entire Body Assessment (REBA) and Rapid Upper Limb Assessment (RULA), but none of these tools are specific to computer work that occurs in office settings [15–17]. OWAS was developed for the steel industry and its risk assessment includes the weight of load handled, entire body posture and frequency of exposure. Sampling occurs using fixed-time intervals for evaluation [17]. The QEC was developed to allow a rapid assessment of work risk factors and includes upper arms, back and neck postures, frequency and duration of posture exposure, force, and workload due to visual demand, driving, use of vibrating tools, difficulties to keep up with work, and stress [17]. REBA assesses entire body posture in healthcare and service industries and its risk assessment includes entire body posture, load handled, and physical activity [17]. RULA has been used to evaluate sitting work [16–22], and although it was modified and validated for use with office workers [23], it does not consider computer peripherals and workers’ interactions with them.

A recent study using the Rapid Office Strain Assessment (ROSA) [24] tool showed that ROSA scores were more highly correlated with musculoskeletal symptoms than RULA scores in computer workers, and that ROSA was easy-to-use for assessing computer workstations [25]. Thus, ROSA may assist in assessing computer-related risk factors when other objective measurements are not available. In addition, the ROSA final score has been shown to be positively associated with musculoskeletal symptoms [24, 26, 27].

ROSA is a validated, practical, easy-to-use and inexpensive observation-based method. It has been used extensively in a variety of countries, and it has been translated into multiple languages [28–30], including Brazilian Portuguese (ROSA-Br) [31]. ROSA scores have been correlated positively with discomfort scores [24], and our group has reported previously on the reliability, internal consistency, construct validity, and accuracy of ROSA’s risk score [31]. The findings of this study support the use of the ROSA-Br for ergonomic field assessments and research. These advantages enable its use in large organizations to screen workstations that require intervention. ROSA can also be used online to help with office ergonomics in work-from-home situations [32]. Assessments can be made by an ergonomist or by workers on their own, and video records can be assessed (for example during the recent pandemic) if ergonomists are unable to enter workspaces in person. Previous studies have demonstrated the versatility and specificity of the ROSA tool for risk assessments of office workstations [24, 32, 33]. Furthermore, to add to the growing body of literature in support of the use of this tool globally, further validation of ROSA as an effective tool for detecting changes in ergonomic risk factors is needed. This tool has never been used to detect ergonomic intervention changes. Further establishing the effectiveness of ROSA as a risk assessment tool for office workstation interventions will help in the ongoing efforts to reduce work-related musculoskeletal discomfort and disorders in the office environment.

Thus, the aim of the present study was to evaluate if ROSA scores reflect changes in risk factors after an ergonomics intervention among office workers. Our hypothesis was that ROSA would be able to identify a reduction in the number and magnitude of risk factors in the group that received the intervention; we expected that the group that did not receive the intervention would not experience a reduction in ROSA scores.

## Methods

### Study design and population

The present study is an intervention study that compares ROSA scores obtained pre and post ergonomic intervention among a group of office workers within the e-learning sector of a public university in the state of São Paulo, Brazil. Ninety-five employees work in this sector within administration, human resources, finances, institutional relations, pedagogical coordination, professional improvement, and innovative technologies in education. The main duties of these workers are performed on computers.

### Participants

All 95 employees were invited to participate and sixty office workers took part in this study. The inclusion criteria were age between 18 and 60 years, work shift duration of at least four hours/day five days/week, and formal agreement to participate. The exclusion criteria were body mass index (BMI) greater than 30 kg/m^2^, being left-handed, not having a fixed workstation or sharing a workstation with a coworker, using a laptop computer or two monitors, and having undergone any kind of surgery in the previous six months. These criteria were applied because some situations might interfere in the subjective results or were not included in the ROSA tool. Failure to complete the evaluations was an exclusion criterion.

The participants were allocated to a control group (*n* = 29) and experimental group (*n* = 31) using a cluster randomization method, with the office (i.e., each room) as the cluster unit. Two buildings with a total of 13 rooms were evaluated. Each office (room) contained a varying number of individuals; all workers belonging to the same room were necessarily included in the same group. The clusters were randomized according to the size, then the proportion of individuals allocated to each group was similar. This method was chosen to avoid contamination between groups, i.e., to prevent the possibility of any worker allocated in the control group to perform modifications on their own, by seeing changes being made in a nearby worker. The control group did not participate in any ergonomic intervention, whereas the experimental group was informed about risk factors related to their workstation and the prevention of musculoskeletal disorders. Adjustments were also made to the workstations of experimental group members.

### Equipment and instruments

A self-administered questionnaire was completed by participants who provided information regarding their sex, age, height, body mass, dominant hand, schooling, occupational history, pain or physical discomfort, and living habits. Anthropometric data (i.e., measurements of eye level, elbow and shoulder height from floor in the seated position; thigh length and height) and workstation dimensions (i.e., seat height and width; table height, width and length; monitor height; distance from the monitor, mouse and keyboard to the front edge of the table) were recorded by an experienced physiotherapist using a tape measure. The physiotherapist had a Master of Science in Ergonomics.

Perceived discomfort was assessed using a visual analogue scale (VAS) measuring 10 cm with verbal anchors on the left indicating “no discomfort” and the right indicating “worst discomfort imaginable” [34]. Each participant was asked to make a vertical mark with a pen on the paper which had a line corresponding to the level of discomfort that he/she felt at the time of evaluation.

### Rapid Office Strain Assessment (ROSA)

The Rapid Office Strain Assessment (ROSA) is an office risk assessment tool. Based on Canadian guidelines and a panel of experts, awkward postures and risk factors related to the use of peripherals during office work (mouse, monitor, keyboard, etc.) were scored to provide information regarding the need for ergonomic intervention [24].

ROSA scoring is based on sections, including the chair (section A), screen and phone (section B), keyboard and mouse (section C) [24]. Risk factors were scored according to each subsection (Table 1).

**Table 1:** ROSA scoring sections and subsections, score range and risk factors [24]

Section and subsections	Score range	Risk factors
Section A	2 to 9	
Armrest and back support	2 to 9	Sum of armrests and back support
Arm rests	1 to 5	Lack of arm support, being too high, presenting hard surface, being too wide or non-adjustable
Back support	1 to 4	Awkward back posture, lack of lumbar support, height of the work surface and non-adjustable back support
Seat pan height/depth	2 to 8	Sum of seat pan height and depth
Seat pan height	1 to 5	Inadequate seat height and insufficient space for legs
Seat pan depth	1 to 3	Inadequate seat depth and non-adjustable seat pan depth
Section B	1 to 9	
Screen	0 to 7	Screen height, screen distance from user, neck awkward posture, presence of glare on screen and lack of document holder
Phone	0 to 6	Phone reach > 30 cm, phone held by neck and shoulder and having no hands-free
Section C	1 to 9	
Keyboard	0 to 7	Awkward wrist postures, keyboard too high and keyboard on non-adjustable platform
Mouse	0 to 7	Long mouse reaching, mouse and keyboard being on different surfaces, mouse pinch grip and the presence of pressure points while mousing

[24] Sonne M, Villalta DL, Andrews DM. Development and evaluation of an office ergonomic risk checklist: ROSA—Rapid Office Strain Assessment. Appl Ergon. 2012;43:98–108

Each subsection has a chart and a corresponding value due to the combination of the partitions. Within Section A scoring, armrests and back support and seat pan height/depth are added together to compose horizontal and vertical axes of Section A chart, respectively. Then, chart values for both sections B and C are combined into another chart, resulting in a monitor and peripheral scores. In a final chart, chair, screen, and peripherals (A, B and C) are combined for risk classification [24].

Photographs were taken (front, back, side, and top views), using a digital camera (resolution 14.1 megapixels, digital zoom 2x, optical zoom 4x, Sony, DSC-W530) with 1.0 to 1.5 m of distance from the worker. The position of the eyes, upper arms, upper and lower back, knees and feet were used as references when the workers' postures were observed directly from the photographs by the assessor. There were no angles and distances measured from the photographs and no landmarks were positioned on the participants [33].

A questionnaire on the use of each workstation component was administered. All procedures were conducted by a single trained physiotherapist to minimize possible variability between evaluators. The final ROSA score ranged from 1 to 10, with a higher total score corresponding to a greater risk of musculoskeletal disorders. A score of five or more points denoted a need for immediate intervention [24].

### Procedures

Anthropometric and workstation measurements were taken in both groups during regular working time. The anthropometric measurements and the photos for ROSA assessment were taken on the same day, before and after the intervention. In the experimental group, participant anthropometric measurements were used to make chair adjustments. Before and after the intervention (experimental group) or pause (control group), photographs were taken for the evaluation of risk factors using ROSA. The observation and photographs lasted about one hour at each evaluation moment. Over this time, on average, eight photographs were taken for each evaluation moment so that the interaction of the worker with all the furniture/peripherals that ROSA contemplates could be captured, besides the need to perform the photographs in all views.

**Fig. 1:**
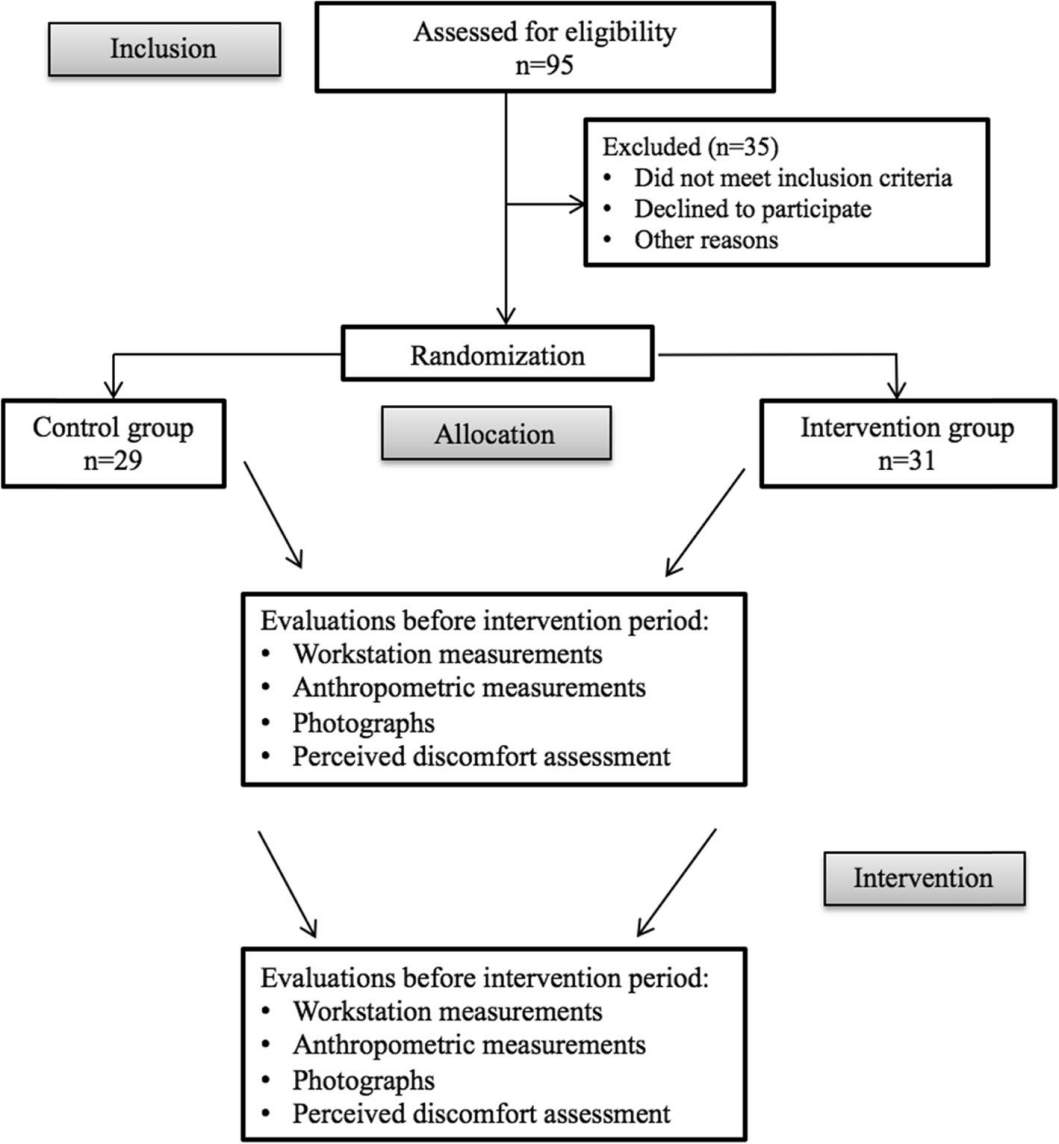
Flowchart of study

Perceived discomfort was assessed on the same day at two moments: 1. before the intervention (or pause), after at least one working hour and 2. after the intervention (or pause), after at least one working hour. The data collection procedures are displayed in Fig. 1.

### Intervention

In the experimental group, the entire workstation was adjusted according to ergonomic recommendations [35, 36] by a single consulting physiotherapist. Since table heights were not adjustable, chair height was adjusted to allow a neutral shoulder posture and elbows flexed at 90° while using the computer. Any chair that did not allow adjustments was replaced with an adjustable chair and a footrest was provided, if necessary.

Monitor height was adjusted so that the viewing level was on the upper third of the screen. The monitor was positioned in front of the worker 40 to 75 cm from the worker’s view [24]. The keyboard and mouse were positioned at a distance that allowed the forearm to be supported on the desk. The mouse was aligned with the shoulder and positioned close to the keyboard [24].

The workstation adjustments were performed in the presence of the workers and the physiotherapist explained the reason for each adjustment. The worker also received advice on the importance of the adjustments and additional ergonomic guidance was offered (Table 2). The intervention took about 10–15 min and it was performed only once. The participants in the control group took a 10-min walking break with no workstation adjustments or orientation. For ethical reasons, the control group received the intervention, as needed, after the post-intervention evaluation.

**Table 2:** The standardized ergonomic guidance provided for the workers

Recommendations for maintaining the proper workplace
• The chair should be adjustable in relation to seat height allowing 90° knee flexion angle and the feet to stay well supported on the floor (or foot rest)
• The seat length should allow weight to be discharged into the ischia and thighs without compressing the popliteal region
• The angle between the seat and the backrest should allow participants to assume a 90° to 120° hip flexion posture
• The backrest should provide support for the lower back
• The table height should correspond to the distance between the elbow and the floor, with the individual sitting in a suitable chair
• It should allow the shoulders to remain relaxed without abduction or flexion
• It should offer enough space for leg movement under the table
• The monitor should be at a distance of 45 to 70 cm to worker’s view
• It should allow for a viewing level in the upper third of the screen

### Data analysis

#### Dependent and independent variables

The dependent variables were the ROSA final and section scores. The independent variables were group (experimental group vs. control group) and time (pre-intervention vs. post-intervention).

### Statistical analysis

The data were analyzed descriptively with measures of central tendency, variability, and confidence intervals. The distribution of quantitative variables was tested using the Shapiro Wilk test. Groups were compared using an independent t-test for normally distributed variables and Mann–Whitney test for not normally distributed variables. For sex, level of education, presence of pain, practice of physical activity during leisure time, smoking and regular drinking, Chi square tests were used. For the ROSA final and section scores, the Mann–Whitney test was used for the inter-group comparison considering the change in scores (post–pre) as the dependent variable. Perceived discomfort was used as an external reference for the effectiveness of the intervention. The change in scores (post–pre) was classified as an improvement (i.e., perceived discomfort decreased over time) or worsening (i.e., perceived discomfort increased over time), disregarding the groups (experimental group or control group). Then, the change in ROSA score was compared using the Mann–Whitney test. Spearman’s rank order correlation was used to test the correlation between ROSA scores and the perceived discomfort. The data were analyzed using SPSS (version 22.0) and the significance level was set at 5% (α = 0.05). Effect sizes (d) were calculated to estimate the magnitude of the difference between groups.

### Sample size

The sample size was defined using the G*Power program (version 3.1). The Mann–Whitney test was considered for the calculation. Considering a 5% significance level (α = 0.05), 80% power (β = 0.20) and a large effect size (0.8), 52 participants were needed in the total sample.

## Results

The personal and demographic data of the two groups are displayed in Table 3. Mean age, body mass, height, BMI, daily working hours and job seniority were similar between groups. Both groups had a larger proportion of women. Although heterogeneity was found with regard to schooling, approximately 26% of both groups had completed a postgraduate degree. The groups had similar proportions of symptomatic participants and workers were classified as physically active, although the experimental group had a slightly higher proportion. The daily consumption of alcohol and cigarettes was low in both groups.

**Table 3:** Personal and demographic data for control group (CG) and experimental group (EG). Data are expressed as means and standard deviations (SD) or total number and percentage (%)

Personal and demographic data	CG (*n* = 29)	EG (*n* = 31)	*P*
Age (years) [mean (SD)]	29.4 (8.4)	28.3 (7.7)	0.65
Body mass (kg) [mean (SD)]	68.2 (14.8)	68.4 (11.4)	0.93
Height (cm) [mean (SD)]	166.7 (10.8)	169.3 (9.6)	0.34
BMI (kg/cm^2^) [mean (SD)]	24.3 (3.6)	23.8 (2.5)	0.49
Daily hours of work (hours) [mean (SD)]	7.1 (1.3)	7.1 (1.4)	0.68
Job seniority (months) [mean (SD)]	28.3 (25.1)	32.7 (26.7)	0.76
Sex [n (%)]			0.08
Female	22 (75.9)	17 (54.8)	
Male	7 (24.1)	14 (45.2)	
Education [n (%)]			0.79
Attending undergraduate school	7 (22.1)	8 (25.8)	
Complete undergraduate	13 (44.9)	12 (38.7)	
Attending graduate school	1 (3.4)	3 (9.7)	
Complete postgraduate degree	8 (27.6)	8 (25.8)	
Presence of pain symptoms [n (%)]	14 (48.3)	16 (51.6)	0.50
Practice of physical activity during leisure [n (%)]	11 (37.9)	18 (58.1)	0.09
Smoking [n (%)]	3 (10.3)	4 (12.9)	0.53
Regular drinking [n (%)]	1 (3.4)	4 (12.9)	0.19

**Fig. 2:**
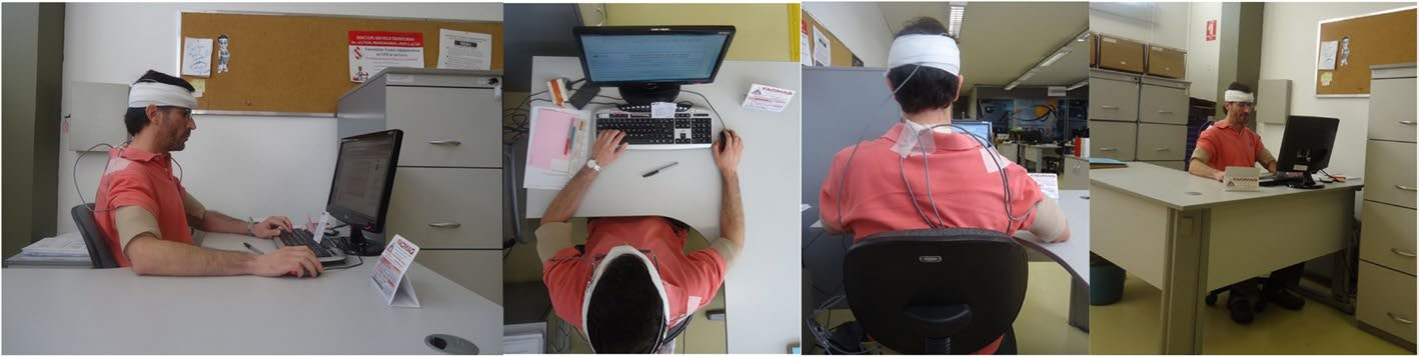
Work position of participants

The ROSA scores before and after the intervention are displayed in Table 4. The ROSA final score was similar between groups before the intervention. The experimental group exhibited a statistically significant reduction in risk factors for all scores after the intervention (P < 0.01). The largest reduction was found for the chair section (A), followed by the mouse and keyboard section (C) and the monitor and telephone section (B), with a mean reduction of 2.9, 1.6 and 0.8 points, respectively. An example of the workplace is presented in Fig. 2.

**Table 4:** Mean (SD) values for Rapid Office Strain Assessment (ROSA) final and section scores in the control group (CG) and experimental group (EG) before (pre) and after (post) intervention

ROSA	CG (*n* = 29)			EG (*n* = 30^a^)			Effect size (*d*)	*P*
	Pre	Post	Variation	Pre	Post	Variation		
Final Score	6.2 (1.2)	6.2 (1.1)	0.0 (0.4)	6.9 (1.3)	3.9 (0.6)	-2.9 (1.4)	2.84	< 0.01
Section A—Chair	6.1 (1.2)	6.2 (1.1)	0.1 (0.4)	6.9 (1.3)	3.9 (0.6)	-2.9 (1.4)	2.84	< 0.01
Height and Pan Depth	4.8 (1.0)	4.9 (1.1)	0.1 (0.3)	5.0 (1.1)	3.2 (0.5)	-1.8 (1.2)	2.26	< 0.01
Armrest and Back Support	5.6 (1.1)	5.7 (1.1)	-0.1 (0.3)	6.3 (1.2)	3.9 (0.6)	-2.4 (1.3)	2.58	< 0.01
Section B—Monitor and Phone	2.7 (1.1)	2.7 (1.0)	-0.1 (0.2)	2.3 (0.9)	1.4 (0.6)	-0.8 (0.8)	1.29	< 0.01
Monitor	3.4 (1.0)	3.4 (1.1)	0.0 (0.2)	3.1 (0.9)	2.1 (0.3)	-0.9 (0.8)	1.69	< 0.01
Phone	1.1 (1.0)	1.1 (1.0)	0.0 (0.0)	0.7 (1.3)	0.5 (0.9)	-0.2 (0.6)	0.58	0.02
Section C – Mouse and Keyboard	4.1 (1.4)	4.2 (1.2)	0.1 (0.2)	4.2 (1.1)	2.6 (0.6)	-1.6 (1.0)	1.89	< 0.01
Mouse	2.8 (0.8)	2.8 (0.8)	0.1 (0.4)	3.1 (0.7)	2.2 (0.5)	-0.8 (0.6)	1.54	< 0.01
Keyboard	3.7 (1.1)	3.8 (0.9)	0.1 (0.4)	3.4 (0.9)	2.1 (0.6)	-1.3 (0.7)	2.34	< 0.01

d = effect size for variation (post–pre scores) between groups; *P*-values refer to comparisons between groups for variation (post–pre scores)

^a^missing data

Considering the variation of the final ROSA score (post–pre scores), in the GC two workers presented a one-point increase, 25 did not present any variation and two workers presented a one-point reduction. On the other hand, in the experimental group, none of the workers had an increase on the final score and two workers showed no variation; about 67% of experimental group workers reduced three or more points after the intervention.

**Fig. 3:**
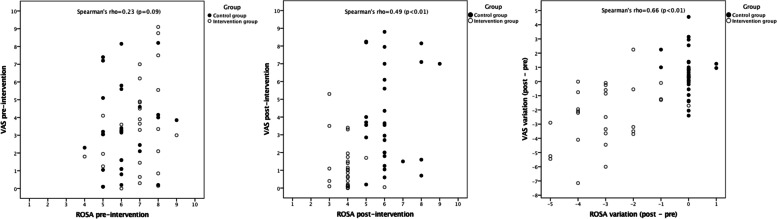
Scatter-plot between ROSA score and perceived discomfort pre- and post-intervention

Regarding discomfort, 23 workers perceived an increase over time (worsening) and 35 workers a decrease over time (improving). The difference between groups for the change in ROSA score between the pre- and post-intervention evaluations was statistically significant (*P* < 0.05). The worsening group showed a mean reduction of 0.09 points (SD = 0.60) in the ROSA score and the improving group showed a mean reduction of 2.31 points (SD = 1.71). There was a significant positive correlation between ROSA scores and discomfort post-intervention (*r* = 0.49; *p* < 0.01) and between changes in ROSA scores and changes in discomfort (*r* = 0.66; *p* < 0.01) (Fig. 3).

## Discussion

In the present study, ROSA detected changes in the experimental group risk factors after the ergonomic intervention. The results also showed that the groups were similar at baseline regarding exposure to risk factors, as 72% of the control group and 80% of the experimental group had ROSA final scores between 6 and 8 points. A significant reduction in all ROSA scores occurred in the experimental group. These results were expected since the ergonomic recommendations were followed by the participants. Thus, changes in the ROSA scores were found, which gives credibility to ROSA as a tool that would be suitable to use for other similar office workstation interventions.

A final ROSA score greater than five [24] or six points [31] indicates an increased risk of musculoskeletal discomfort and the need for immediate intervention. The workers who participated in the present study had never received any kind of ergonomic training, which explains the high scores obtained before the intervention. Chairs that did not allow adjustments also explain the higher initial scores.

Sonne et al. [24] found a mean final score of 4.13 points among 72 office workers. These results are similar to those of the present study after the intervention in the experimental group. The high ROSA scores at baseline may be related to the different types of furniture found in Brazilian offices. Another study conducted in Brazil found a mean of 5.83 on the final ROSA score [31], which is very similar to the present study. In other countries, adjustable tables and chairs are more common than in Brazil.

In both groups, the chair section contributed most to the high ROSA scores. Since table height could not be adjusted, workers often raise the chair height to obtain an adequate view of the screen. Thus, the chair heights of short stature workers were classified as being too high. Other reasons for the high ROSA scores in section A were the insufficient space under the table and non-adjustable chair pan depth and backrest.

Regarding perceived discomfort, this subjective measure was used as an external reference to demonstrate the ability of the ROSA to detect the effect of the intervention. We found that workers who perceived more discomfort also showed a lower reduction in ROSA score and vice-versa. Thus, the findings obtained in the present study reinforce the notion that workplace interventions can reduce the exposure to risk factors and, consequently, reduce perceived discomfort among office workers.

Photographs were used in the present study to obtain the ROSA score, which contributes to the versatility of ROSA use by practitioners in cases for which scoring ROSA at the workplace is not possible. The variability in ROSA scores throughout a given day is usually minimal, as this tool assesses risk factors based on the interaction between the worker and the workstation components and does not rely heavily on quantifying body postures, such as OWAS or RULA, for example. Of course, if the components of the workstation (e.g., chair, monitor, mouse or keyboard) are changed during the work day, the variability of ROSA scores would increase.

**Fig. 4:**
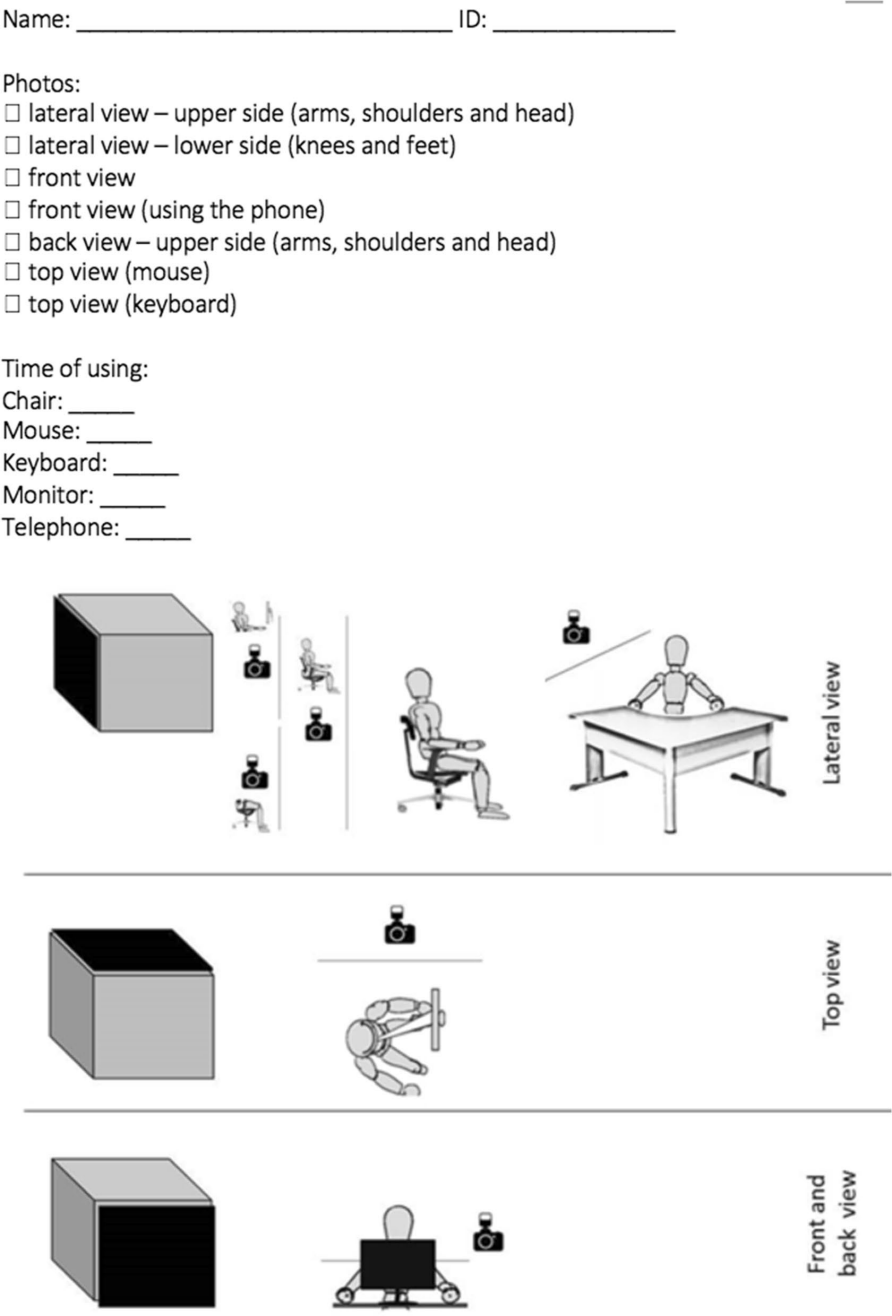
Recommendation for checklist and photographs for scoring ROSA

This method also enables checking whether the workers are following the advice given by the physiotherapist. Moreover, a recent study reports that photo-based assessments are valid and practical and suggests that photos be taken from an orthogonal perspective [33]. We also recommend taking several views (Fig. 4). For the side view, one photo should be on the upper portion of the body, focusing on the arms, shoulders and head, and another photo should focus on the knees and feet. Front, back and top view photos are also necessary. These recommendations could also be provided for workers who are working from home due to COVID-19 pandemic restrictions.

### Limitations

This study has some limitations that should be addressed. We could not assess the responsiveness of the ROSA tool because we did not use an external anchor, for example, a global improvement perception scale.

The type of furniture and other workstation components were not the same for all workers before the intervention. Therefore, some workers did not have a high ROSA score on the pre-test evaluation. The assessor was not blinded to the treatment allocation, as it was the same assessor who provided the intervention. The scoring conducted by photos, and consequently the presence of the physiotherapist in the workplace, may have had a supervisory effect.

There are also intrinsic limitations of the chosen scoring system, since it is based on subjective measures of the assessor, and not on technologies such as 3D cameras for measuring joint angles and distances. Thus, the expertise of the assessors is relevant, and it is possible that different assessors may achieve different outcomes. On the other hand, using the same assessor would increase the reliability of the measurements.

ROSA is based on static postures of different body regions, and does not consider the joint loads and/or muscle activations that would help to quantify overload and fatigue. Possible additional instrumentation usable for testing different types of ergonomic interventions could be represented by an ergonomic evaluation based on sEMG data, as suggested in different works [37, 38]. However, the study team was in the work environment for some time and workers did become accustomed to its presence. One of the strengths of this study is that it was performed in a working environment. Field-based evaluations such as this are critical for improving the external validity and assessing the effectiveness of ergonomic risk assessment tools such as ROSA.

### Practical recommendations and perspectives on the use of ROSA

The researchers perceived some practical issues during the ROSA scoring. The instructions do not mention how the evaluator should score if a chair is too high, but the worker uses a footrest to compensate. In the present study, footrest height was considered when scoring the chair section.

ROSA quantifies risk based on the final score, but this score is the result of the intersection between the chair and monitor and peripheral sections. For instance, a final score of 5 points, which suggests the need for an immediate intervention, is reached by a score of 5 on the chair section and a score between 1 and 5 on the monitor and peripheral sections, or vice versa. Therefore, the final value alone can underestimate the influence of furniture other than the chair and its role in the occurrence of musculoskeletal symptoms.

Therefore, a possible change to ROSA would be to include a cutoff score for each section to facilitate intervention proposals. Moreover, this aspect underscores the need to present the individual section scores in studies that use ROSA. Separate scores would enable a better understanding of the work situation and a more focused intervention. Moreover, if one of the peripherals is not commonly used, such as the phone, its individual effect could be seen relative to the other section and final scores.

Another suggestion that may make it easier to use this instrument is to include an option in which the phone is not used. In the present study, this peripheral received a zero score (0). A zero score occurs on section B (monitor and telephone) sub-item telephone when the worker uses a headset or one hand on the phone and a neutral neck posture (score 1) and the duration is less than one hour per day intermittently or less than 30 min consecutively, as this exposure determines the subtraction of 1 point.

Arm support could also be described in more detail for ROSA users. This risk factor can be identified in the sub-items armrests, back support, and keyboard. It is not clear how to score if the worker without armrests supports their forearms on the table. Thus, there may be an overestimation of some office risk factors.

Some difficulties were encountered regarding the determination of working time using each peripheral. As most workers have an eight-hour workday, observation during this period may become unfeasible. One option is to ask workers how much time they remain seated, how long they use the components of their workstation (e.g., monitor, keyboard, etc.) while working. However, self-reports may not be reliable [39], especially for the mouse, which is a peripheral that is not used alone, but together with the monitor. Thus, we recommend quantifying the time of use of each peripheral rather than relying on workers’ perceptions. Moreover, the perception of duration depends on the activity, symptoms, stress level or other external factors [40]. Another suggestion is for the tool to consider musculoskeletal symptoms, with the addition of points to the final score if the worker reports pain during the use of each peripheral. The description of ROSA could also include a guide on how to evaluate workers who use laptops or two monitors.

Thus, future studies could address the validation of the ROSA method, comparing ROSA area and final scores with the development of musculoskeletal discomfort, pain, or diagnosed musculoskeletal disorders. Also, the prospective follow-up of workers who received ergonomic interventions could show the benefits of ROSA score reduction on a longitudinal basis.

## Conclusion

The Rapid Office Strain Assessment identified a reduction in risk factors after the ergonomic intervention. Thus, ROSA scores reflected changes in risk factors after ergonomic intervention among office workers. Consequently, it is suggested that this tool can be used for ergonomic screening, recommending workstation modifications, and testing the effectiveness of ergonomic interventions, thereby contributing to evidence-based ergonomics programs. The relevance of this tool increased due to the COVID-19 pandemic, as many workers transitioned to home offices but still require ergonomics evaluations. The use of video cameras in this study, in addition to previous research using ROSA which relied on self-assessments [32], and photograph-based assessments [33] provide a comprehensive way of using the tool to gain insight into office ergonomics risk without an ergonomist having to make their way onsite. This reduces risk of exposure for both the person conducting the ergonomics assessment as well as receiving the ergonomics assessment. However, practical recommendations need to be stressed in future ergonomics studies to further establish good clinimetric properties of the ROSA.

## Data Availability

The datasets used and/or analysed during the current study available from the corresponding author on reasonable request.
